# 1-[(1,3-Dithio­lan-2-yl)meth­yl]-6-methyl-8-nitro-1,2,3,5,6,7-hexa­hydro­imidazo[1,2-*c*]pyrimidine

**DOI:** 10.1107/S160053681003206X

**Published:** 2010-08-18

**Authors:** Zhongzhen Tian, Haijun Dong, Dongmei Li, Gaolei Wang

**Affiliations:** a Shandong Provincial Key Laboratory of Fluorine Chemistry and Chemical Materials, School of Chemistry and Chemical Engineering, University of Jinan, People’s Republic of China; b School of Sciences, University of Jinan, People’s Republic of China

## Abstract

In the title compound, C_11_H_18_N_4_O_2_S_2_, the dithiol­ane ring displays an envelope conformation, the tetra­hydro­pyrimidine ring has a conformation that is between half-chair and screw-boat, and the imidazole ring is essentially planar (r.m.s. deviation = 0.0017 Å). No significant directional inter­molecular inter­actions are present in the structure.

## Related literature

For related structures, see: Tian *et al.* (2009 ▶). For background to neonicotinoid insecticides, see Mori *et al.* (2001 ▶); Kagabu (1997 ▶); Tian *et al.* (2007 ▶).
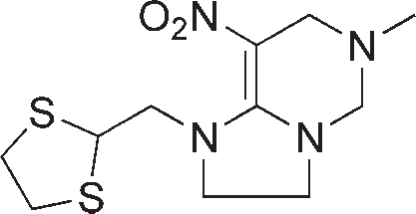


## Experimental

### 

#### Crystal data

C_11_H_18_N_4_O_2_S_2_
*M* *_r_* = 302.41
Triclinic, 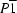

*a* = 8.0326 (7) Å
*b* = 9.3521 (8) Å
*c* = 10.1109 (9) Å
α = 80.461 (1)°
β = 83.497 (1)°
γ = 68.043 (1)°
*V* = 693.62 (10) Å^3^
*Z* = 2
Mo *K*α radiation
μ = 0.39 mm^−1^
*T* = 293 K
0.26 × 0.23 × 0.18 mm

#### Data collection

Bruker APEXII CCD area-detector diffractometer
Absorption correction: multi-scan (*SADABS*; Bruker, 2005 ▶) *T*_min_ = 0.906, *T*_max_ = 0.934
7993 measured reflections
3178 independent reflections
2826 reflections with *I* > 2σ(*I*)
*R*_int_ = 0.020

#### Refinement

*R*[*F* ^2^ > 2σ(*F* ^2^)] = 0.037
*wR*(*F* ^2^) = 0.098
*S* = 1.06
3178 reflections
173 parameters
H-atom parameters constrained
Δρ_max_ = 0.38 e Å^−3^
Δρ_min_ = −0.34 e Å^−3^

### 

Data collection: *APEX2* (Bruker, 2005 ▶); cell refinement: *SAINT* (Bruker, 2005 ▶); data reduction: *SAINT*; program(s) used to solve structure: *SIR97* (Altomare *et al.*, 1999 ▶); program(s) used to refine structure: *SHELXL97* (Sheldrick, 2008 ▶); molecular graphics: *SHELXTL* (Sheldrick, 2008 ▶); software used to prepare material for publication: *WinGX* (Farrugia, 1999 ▶).

## Supplementary Material

Crystal structure: contains datablocks I, global. DOI: 10.1107/S160053681003206X/zl2297sup1.cif

Structure factors: contains datablocks I. DOI: 10.1107/S160053681003206X/zl2297Isup2.hkl

Additional supplementary materials:  crystallographic information; 3D view; checkCIF report

## Figures and Tables

**Fig. 1.**
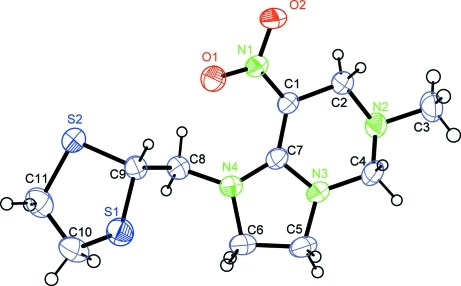
The molecular structure of the title compound with the atom numbering scheme. Displacement ellipsoids are drawn at the 50% probability level. The H atoms are shown as spheres of arbitrary size.

## supplementary crystallographic information

### Comment

Imidacloprid, a commercially sold insecticide modeled after nicotine, gains its activity by acting on the nicotinic acetylcholine receptor (nAChR) of insect neuronal systems (Mori *et al.*, 2001). Imidacloprid and other neonicotinoid insecticides have become a major insecticide class with high activities and are widely used for crop protection and veterinary pest control (Kagabu, 1997). Our interest was introducing sulfur atoms into the lead struture and synthesizing a series of new compounds, in which the title compound exhibited moderate insecticidal activities against pea aphids.

The structure of the title compound is shown in Fig. 1 with the atom-numbering scheme. The dithiolane ring displays a typical envelope conformation. The nitro group is almost coplanar with the olefin-amine plane [C7—C1—N1—O2 = 173.30 (14)°]. Around N3 and N4 the sums of the angles are 353.32° and 349.31°, respectively, indicating that they are nearly *sp*^2^ hybridized and leading to an essentially planar imidazole ring. The N2 atom exhibits a hybridization close to *sp*^3^ with C—N—C angles between 109.41 (13)° and 110.33 (14)°. The tetrahydropyrimidine ring has a conformation that is in between half-chair and screw-boat. No significant directional intermolecular interactions are present in the structure and the packing is dominated by van der Waals forces.

### Experimental

The title compound was synthesized according to the literature (Tian *et al.*, 2007). Single crystals suitable for X-ray analysis were obtained by slow evaporation of a solution of dichloromethane and ethyl acetate of the title compound.

### Refinement

All H atoms were placed in their calculated positions and then refined using a riding model with C—H = 0.95–0.99 Å, *U*_iso_(H) = 1.2 (1.5 for methyl groups) times *U*_eq_(C).

### Crystal data

**Table:**

C_11_H_18_N_4_O_2_S_2_	*Z* = 2
*M**_r_* = 302.41	*F*(000) = 320
Triclinic, *P*1	*D*_x_ = 1.448 Mg m^−^^3^
Hall symbol: -P 1	Mo *K*α radiation, λ = 0.71073 Å
*a* = 8.0326 (7) Å	Cell parameters from 4541 reflections
*b* = 9.3521 (8) Å	θ = 2.4–27.7°
*c* = 10.1109 (9) Å	µ = 0.39 mm^−^^1^
α = 80.461 (1)°	*T* = 293 K
β = 83.497 (1)°	Prism, colourless
γ = 68.043 (1)°	0.26 × 0.23 × 0.18 mm
*V* = 693.62 (10) Å^3^	

### Data collection

**Table:**

Bruker APEXII CCD area-detector diffractometer	3178 independent reflections
Radiation source: fine-focus sealed tube	2826 reflections with *I* > 2σ(*I*)
graphite	*R*_int_ = 0.020
φ and ω scans	θ_max_ = 27.7°, θ_min_ = 2.1°
Absorption correction: multi-scan (*SADABS*; Bruker, 2005)	*h* = −10→10
*T*_min_ = 0.906, *T*_max_ = 0.934	*k* = −12→11
7993 measured reflections	*l* = −13→13

### Refinement

**Table:**

Refinement on *F*^2^	Primary atom site location: structure-invariant direct methods
Least-squares matrix: full	Secondary atom site location: difference Fourier map
*R*[*F*^2^ > 2σ(*F*^2^)] = 0.037	Hydrogen site location: inferred from neighbouring sites
*wR*(*F*^2^) = 0.098	H-atom parameters constrained
*S* = 1.06	*w* = 1/[σ^2^(*F*_o_^2^) + (0.0447*P*)^2^ + 0.2729*P*] where *P* = (*F*_o_^2^ + 2*F*_c_^2^)/3
3178 reflections	(Δ/σ)_max_ < 0.001
173 parameters	Δρ_max_ = 0.38 e Å^−^^3^
0 restraints	Δρ_min_ = −0.34 e Å^−^^3^

### Special details

**Table:**

Geometry. All e.s.d.'s (except the e.s.d. in the dihedral angle between two l.s. planes) are estimated using the full covariance matrix. The cell e.s.d.'s are taken into account individually in the estimation of e.s.d.'s in distances, angles and torsion angles; correlations between e.s.d.'s in cell parameters are only used when they are defined by crystal symmetry. An approximate (isotropic) treatment of cell e.s.d.'s is used for estimating e.s.d.'s involving l.s. planes.
Refinement. Refinement of *F*^2^ against ALL reflections. The weighted *R*-factor *wR* and goodness of fit *S* are based on *F*^2^, conventional *R*-factors *R* are based on *F*, with *F* set to zero for negative *F*^2^. The threshold expression of *F*^2^ > σ(*F*^2^) is used only for calculating *R*-factors(gt) *etc*. and is not relevant to the choice of reflections for refinement. *R*-factors based on *F*^2^ are statistically about twice as large as those based on *F*, and *R*- factors based on ALL data will be even larger.

### Fractional atomic coordinates and isotropic or equivalent isotropic displacement parameters (Å^2^)

**Table:**

	*x*	*y*	*z*	*U*_iso_*/*U*_eq_	
C1	0.92947 (18)	0.16249 (17)	0.65277 (14)	0.0319 (3)	
C2	1.0078 (2)	0.10338 (19)	0.78875 (16)	0.0375 (3)	
H2A	0.9189	0.1516	0.8570	0.045*	
H2B	1.0364	−0.0081	0.8074	0.045*	
C3	1.2634 (3)	0.0458 (2)	0.9163 (2)	0.0549 (5)	
H3A	1.2903	−0.0628	0.9137	0.082*	
H3B	1.1876	0.0770	0.9949	0.082*	
H3C	1.3731	0.0636	0.9189	0.082*	
C4	1.2876 (2)	0.0997 (2)	0.67697 (17)	0.0409 (4)	
H4A	1.3993	0.1141	0.6851	0.049*	
H4B	1.3154	−0.0077	0.6650	0.049*	
C5	1.2874 (2)	0.2345 (2)	0.43686 (17)	0.0459 (4)	
H5A	1.3595	0.1393	0.4003	0.055*	
H5B	1.3635	0.2914	0.4466	0.055*	
C6	1.1325 (2)	0.3324 (3)	0.35069 (19)	0.0544 (5)	
H6A	1.1106	0.4422	0.3466	0.065*	
H6B	1.1554	0.3050	0.2602	0.065*	
C7	1.02457 (19)	0.22041 (17)	0.54533 (14)	0.0316 (3)	
C8	0.7976 (2)	0.40401 (18)	0.38887 (15)	0.0361 (3)	
H8A	0.8020	0.5074	0.3646	0.043*	
H8B	0.7182	0.4057	0.4688	0.043*	
C9	0.7185 (2)	0.36571 (18)	0.27473 (15)	0.0369 (3)	
H9	0.7140	0.2614	0.3005	0.044*	
C10	0.7343 (3)	0.5774 (2)	0.0715 (2)	0.0580 (5)	
H10A	0.7905	0.6289	0.1179	0.070*	
H10B	0.7517	0.6059	−0.0244	0.070*	
C11	0.5376 (3)	0.6301 (2)	0.1105 (2)	0.0553 (5)	
H11A	0.4723	0.6319	0.0349	0.066*	
H11B	0.4967	0.7350	0.1334	0.066*	
N1	0.78451 (16)	0.12503 (15)	0.63650 (13)	0.0351 (3)	
N2	1.17052 (18)	0.13662 (16)	0.79623 (13)	0.0379 (3)	
N3	1.19480 (17)	0.20279 (17)	0.56373 (13)	0.0392 (3)	
N4	0.97815 (17)	0.29393 (16)	0.42032 (13)	0.0371 (3)	
O1	0.71913 (17)	0.14805 (16)	0.52435 (12)	0.0489 (3)	
O2	0.72085 (16)	0.06008 (15)	0.73842 (12)	0.0468 (3)	
S1	0.83905 (6)	0.36969 (6)	0.11346 (4)	0.04884 (14)	
S2	0.49010 (6)	0.50283 (6)	0.25217 (5)	0.05506 (15)	

### Atomic displacement parameters (Å^2^)

**Table:**

	*U*^11^	*U*^22^	*U*^33^	*U*^12^	*U*^13^	*U*^23^
C1	0.0265 (6)	0.0378 (7)	0.0330 (7)	−0.0138 (6)	−0.0029 (5)	−0.0035 (6)
C2	0.0335 (7)	0.0431 (8)	0.0374 (8)	−0.0172 (6)	−0.0063 (6)	0.0011 (6)
C3	0.0567 (11)	0.0614 (11)	0.0527 (11)	−0.0299 (9)	−0.0264 (9)	0.0123 (8)
C4	0.0278 (7)	0.0460 (9)	0.0500 (9)	−0.0147 (6)	−0.0088 (6)	−0.0018 (7)
C5	0.0339 (8)	0.0632 (11)	0.0447 (9)	−0.0253 (8)	0.0054 (7)	−0.0052 (8)
C6	0.0422 (9)	0.0823 (13)	0.0432 (9)	−0.0350 (9)	−0.0002 (7)	0.0080 (9)
C7	0.0273 (6)	0.0357 (7)	0.0345 (7)	−0.0137 (6)	−0.0021 (5)	−0.0063 (6)
C8	0.0357 (7)	0.0391 (8)	0.0330 (7)	−0.0133 (6)	−0.0027 (6)	−0.0035 (6)
C9	0.0377 (8)	0.0389 (8)	0.0347 (7)	−0.0155 (6)	−0.0057 (6)	−0.0001 (6)
C10	0.0654 (12)	0.0611 (12)	0.0487 (10)	−0.0323 (10)	−0.0003 (9)	0.0097 (9)
C11	0.0624 (12)	0.0449 (10)	0.0529 (11)	−0.0161 (9)	−0.0072 (9)	0.0039 (8)
N1	0.0296 (6)	0.0421 (7)	0.0368 (6)	−0.0173 (5)	−0.0016 (5)	−0.0039 (5)
N2	0.0367 (7)	0.0424 (7)	0.0384 (7)	−0.0187 (6)	−0.0115 (5)	0.0009 (5)
N3	0.0281 (6)	0.0519 (8)	0.0399 (7)	−0.0200 (6)	−0.0026 (5)	0.0010 (6)
N4	0.0312 (6)	0.0487 (7)	0.0322 (6)	−0.0179 (6)	−0.0007 (5)	−0.0005 (5)
O1	0.0481 (7)	0.0701 (8)	0.0408 (6)	−0.0360 (6)	−0.0129 (5)	0.0011 (6)
O2	0.0402 (6)	0.0648 (8)	0.0420 (6)	−0.0314 (6)	0.0006 (5)	0.0030 (5)
S1	0.0490 (3)	0.0596 (3)	0.0356 (2)	−0.0154 (2)	−0.00023 (17)	−0.01182 (18)
S2	0.0342 (2)	0.0736 (3)	0.0498 (3)	−0.0182 (2)	−0.00542 (18)	0.0116 (2)

### Geometric parameters (Å, °)

**Table:**

C1—N1	1.3686 (18)	C6—H6B	0.9700
C1—C7	1.408 (2)	C7—N3	1.3455 (18)
C1—C2	1.509 (2)	C7—N4	1.3552 (19)
C2—N2	1.4642 (19)	C8—N4	1.4640 (19)
C2—H2A	0.9700	C8—C9	1.529 (2)
C2—H2B	0.9700	C8—H8A	0.9700
C3—N2	1.463 (2)	C8—H8B	0.9700
C3—H3A	0.9600	C9—S1	1.8019 (16)
C3—H3B	0.9600	C9—S2	1.8169 (16)
C3—H3C	0.9600	C9—H9	0.9800
C4—N3	1.444 (2)	C10—C11	1.495 (3)
C4—N2	1.448 (2)	C10—S1	1.802 (2)
C4—H4A	0.9700	C10—H10A	0.9700
C4—H4B	0.9700	C10—H10B	0.9700
C5—N3	1.452 (2)	C11—S2	1.8068 (19)
C5—C6	1.508 (2)	C11—H11A	0.9700
C5—H5A	0.9700	C11—H11B	0.9700
C5—H5B	0.9700	N1—O1	1.2559 (17)
C6—N4	1.488 (2)	N1—O2	1.2641 (17)
C6—H6A	0.9700		
			
N1—C1—C7	123.33 (13)	N4—C8—H8A	108.8
N1—C1—C2	114.77 (12)	C9—C8—H8A	108.8
C7—C1—C2	120.49 (12)	N4—C8—H8B	108.8
N2—C2—C1	112.09 (12)	C9—C8—H8B	108.8
N2—C2—H2A	109.2	H8A—C8—H8B	107.7
C1—C2—H2A	109.2	C8—C9—S1	115.71 (11)
N2—C2—H2B	109.2	C8—C9—S2	109.68 (11)
C1—C2—H2B	109.2	S1—C9—S2	106.91 (8)
H2A—C2—H2B	107.9	C8—C9—H9	108.1
N2—C3—H3A	109.5	S1—C9—H9	108.1
N2—C3—H3B	109.5	S2—C9—H9	108.1
H3A—C3—H3B	109.5	C11—C10—S1	110.59 (13)
N2—C3—H3C	109.5	C11—C10—H10A	109.5
H3A—C3—H3C	109.5	S1—C10—H10A	109.5
H3B—C3—H3C	109.5	C11—C10—H10B	109.5
N3—C4—N2	107.66 (13)	S1—C10—H10B	109.5
N3—C4—H4A	110.2	H10A—C10—H10B	108.1
N2—C4—H4A	110.2	C10—C11—S2	111.27 (13)
N3—C4—H4B	110.2	C10—C11—H11A	109.4
N2—C4—H4B	110.2	S2—C11—H11A	109.4
H4A—C4—H4B	108.5	C10—C11—H11B	109.4
N3—C5—C6	101.87 (13)	S2—C11—H11B	109.4
N3—C5—H5A	111.4	H11A—C11—H11B	108.0
C6—C5—H5A	111.4	O1—N1—O2	120.14 (12)
N3—C5—H5B	111.4	O1—N1—C1	122.24 (13)
C6—C5—H5B	111.4	O2—N1—C1	117.56 (12)
H5A—C5—H5B	109.3	C4—N2—C3	110.33 (14)
N4—C6—C5	103.53 (13)	C4—N2—C2	110.28 (12)
N4—C6—H6A	111.1	C3—N2—C2	109.41 (13)
C5—C6—H6A	111.1	C7—N3—C4	120.45 (13)
N4—C6—H6B	111.1	C7—N3—C5	110.88 (13)
C5—C6—H6B	111.1	C4—N3—C5	122.79 (13)
H6A—C6—H6B	109.0	C7—N4—C8	123.79 (12)
N3—C7—N4	110.27 (13)	C7—N4—C6	108.50 (12)
N3—C7—C1	117.58 (13)	C8—N4—C6	117.02 (13)
N4—C7—C1	132.15 (13)	C10—S1—C9	93.94 (8)
N4—C8—C9	113.81 (13)	C11—S2—C9	98.01 (8)
			
N1—C1—C2—N2	175.52 (13)	N4—C7—N3—C5	−13.07 (19)
C7—C1—C2—N2	8.6 (2)	C1—C7—N3—C5	166.80 (14)
N3—C5—C6—N4	−20.74 (19)	N2—C4—N3—C7	−49.50 (19)
N1—C1—C7—N3	−157.53 (14)	N2—C4—N3—C5	159.98 (14)
C2—C1—C7—N3	8.2 (2)	C6—C5—N3—C7	21.40 (19)
N1—C1—C7—N4	22.3 (3)	C6—C5—N3—C4	174.39 (16)
C2—C1—C7—N4	−171.97 (15)	N3—C7—N4—C8	−144.85 (14)
N4—C8—C9—S1	−62.50 (16)	C1—C7—N4—C8	35.3 (2)
N4—C8—C9—S2	176.50 (10)	N3—C7—N4—C6	−1.68 (19)
S1—C10—C11—S2	28.7 (2)	C1—C7—N4—C6	178.48 (17)
C7—C1—N1—O1	−4.1 (2)	C9—C8—N4—C7	−127.28 (15)
C2—C1—N1—O1	−170.58 (14)	C9—C8—N4—C6	92.38 (18)
C7—C1—N1—O2	173.30 (14)	C5—C6—N4—C7	14.7 (2)
C2—C1—N1—O2	6.8 (2)	C5—C6—N4—C8	160.67 (14)
N3—C4—N2—C3	−174.80 (13)	C11—C10—S1—C9	−42.36 (16)
N3—C4—N2—C2	64.22 (16)	C8—C9—S1—C10	−82.86 (13)
C1—C2—N2—C4	−44.99 (17)	S2—C9—S1—C10	39.62 (10)
C1—C2—N2—C3	−166.51 (14)	C10—C11—S2—C9	−0.91 (17)
N4—C7—N3—C4	−166.78 (14)	C8—C9—S2—C11	99.63 (12)
C1—C7—N3—C4	13.1 (2)	S1—C9—S2—C11	−26.54 (10)
